# Study on potential differentially expressed genes in stroke by bioinformatics analysis

**DOI:** 10.1007/s10072-021-05470-1

**Published:** 2021-07-27

**Authors:** Xitong Yang, Pengyu Wang, Shanquan Yan, Guangming Wang

**Affiliations:** Genetic Testing Center, The First Affiliated Hospital of Dali University, Dali, 671000 Yunnan China

**Keywords:** Bioinformatics analysis, Differentially expressed genes, MicroRNAs, Stroke

## Abstract

Stroke is a sudden cerebrovascular circulatory disorder with high morbidity, disability, mortality, and recurrence rate, but its pathogenesis and key genes are still unclear. In this study, bioinformatics was used to deeply analyze the pathogenesis of stroke and related key genes, so as to study the potential pathogenesis of stroke and provide guidance for clinical treatment. Gene Expression profiles of GSE58294 and GSE16561 were obtained from Gene Expression Omnibus (GEO), the differentially expressed genes (DEGs) were identified between IS and normal control group. The different expression genes (DEGs) between IS and normal control group were screened with the GEO2R online tool. The Gene Ontology (GO) and Kyoto Encyclopedia of Genes and Genomes (KEGG) pathway enrichment analyses of the DEGs were performed. Using the Database for Annotation, Visualization and Integrated Discovery (DAVID) and gene set enrichment analysis (GSEA), the function and pathway enrichment analysis of DEGS were performed. Then, a protein–protein interaction (PPI) network was constructed via the Search Tool for the Retrieval of Interacting Genes (STRING) database. Cytoscape with CytoHubba were used to identify the hub genes. Finally, NetworkAnalyst was used to construct the targeted microRNAs (miRNAs) of the hub genes. A total of 85 DEGs were screened out in this study, including 65 upward genes and 20 downward genes. In addition, 3 KEGG pathways, cytokine − cytokine receptor interaction, hematopoietic cell lineage, B cell receptor signaling pathway, were significantly enriched using a database for labeling, visualization, and synthetic discovery. In combination with the results of the PPI network and CytoHubba, 10 hub genes including CEACAM8, CD19, MMP9, ARG1, CKAP4, CCR7, MGAM, CD79A, CD79B, and CLEC4D were selected. Combined with DEG-miRNAs visualization, 5 miRNAs, including hsa-mir-146a-5p, hsa-mir-7-5p, hsa-mir-335-5p, and hsa-mir-27a- 3p, were predicted as possibly the key miRNAs. Our findings will contribute to identification of potential biomarkers and novel strategies for the treatment of ischemic stroke, and provide a new strategy for clinical therapy.

Stroke is a sudden disorder of cerebral blood circulation, including two types: ischemic stroke (IS) and hemorrhagic stroke [1]. Constituting around 80% of all strokes, ischemic stroke is the most common type of stroke so far. [2]. Stroke has seriously affected people’s living standards and quality of life. A series of complications caused by stroke will not only bring pain to patients, but also increase the burden on families and society. In China, stroke is the second cause of death for residents and one of the main causes of disability for adults [3]. Stroke is considered one of the most devastating neurological disorders, which often results in a combination of physical, behavioral, cognitive, and psychological impairments [4]. The pathophysiologyical mechanism of stroke is complex and involves many aspects, including excitotoxicity, inflammation, oxidative damage, ion imbalance, apoptosis, angiogenesis, and neuroprotection. The most commonly used treatment for stroke is intravenous thrombolysis by using recombinant tissue plasminogen activator (rTPA). However, the biggest disadvantage of this treatment scheme is that the treatment time window is only 3 h [5]. Therefore, it is particularly important to identify molecular targets for effective treatment of stroke and clarify the mechanism of brain injury.

IS is a disease mediated by many mechanisms and pathways. Studies have shown that abnormal gene expression caused by exogenous injury plays an important role in the occurrence and development of IS [6, 7]. The biological processes that the IS injury-related genes participate in and the specific mechanism of IS injury are still unclear. Bioinformatics analysis can use high-throughput gene sequencing technology to analyze the genome, transcriptome, and proteome information of organisms, and can reveal the mechanism of disease occurrence and development from various molecular levels, providing direction for laboratory and clinical research [8]. The biological functions of IS-related genes were analyzed. With the help of bioinformatics methods, differentially expressed genes (DEGs) were screened, and their functions and signal pathways were analyzed. Then, a gene network diagram was constructed and key gene targets were screened. By analyzing the biological functions related to IS, it could lay a foundation for the clinical diagnosis and treatment of IS.

Two original datasets were selected to screen the DEGs between IS sample and normal control group. In order to evaluate the potential molecular mechanism of regulating IS metastasis, DEGs was further analyzed by Gene Ontology (GO) and Kyoto Encyclopedia of Genes and Genomes (KEGG) pathway analysis based on Database for Annotation, Visualization and Integrated Discovery (DAVID) and gene set enrichment analysis (GSEA) database. By constructing PPI network and using the Search Tool for the Retrieval of Interacting Genes (STRING) database and Cytoscape software, a key module was then screened out from the whole network, and the hub genes were identified based on the key module. This study identified several potentially critical stroke-associated biomarkers involved in the progress of IS, which may provide novel insights for exploring the pathogenesis of IS, to understand the pathogenesis and clinical treatment of IS lay the foundation.

## Materials and methods

### Microarray data

The microarray datasets (GSE58294, GSE16561) of IS and control samples were collected from the GEO databade ( https://www.ncbi.nlm.nih.gov/geo/). The author, year, platform, and the proportions of IS and control samples in each dataset were extracted and evaluated. Table 1 gives the details of expression spectrum datasets.

**Table 1:** Details for GEO IS data

Author	Accession	Platform	Samples (nomal/IS sample)
Barr	GSE16561	GPL6883	24/39
Stamova	GSE58294	GPL570	23/69

### Differentially expressed Gene Identification

GEO2R (http://www.ncbi.nlm.nih.gov/geo/geo2r) was applied to perform DEGs analysis between serum samples from IS and control groups, and corrected *P*-value calculations to obtain |log_2_FC|. Genes with correcting *P*-value < 0.05 and |log_2_FC|≥ 0.5 were deemed as DEGs. An online visualization software Funrich (http://funrich.org/) was used to generate the Venn diagram of DEGs. DEGs with logFC > 0 were considered as upregulated genes, while those with logFC < 0 were classified as downregulated genes.

### Functional enrichment analysis

Based on the DAVID database4 (Version 6.8), we carried out GO and KEGG pathway enrichment analyses for the DEGs [9, 10], and the GO analysis included the following domains: biological process (BP), cellular component (CC), and molecular function (MF). A* p* value < 0.05 was specified for statistical significance.

### PPI network construction and module analysis

The PPI network of DEGS was constructed by String V10 online tool (Search Tools for the Retrieval of Interacting Genes, STRING(https://string-db.org/). Used the plug-in of Cytohubba in Cytoscape to calculate the topological structure of PPI network. According to the centrality score, the key nodes in PPI network were determined, and then the key pathogenic genes were deduced.

### MiRNAs associated with hub genes

Used NetworkAnalyst 3.0 (https://www.networkanalyst.ca/), a visual online platform for discovering miRNA-gene interactions in Gene Regulatory Networks, the top 10 central genes were mapped to their corresponding microRNAs, and the hub genes and miRNA were plotted by Cytoscape 3.7.2.

## Results

### IS DEGs identification

**Fig. 1:**
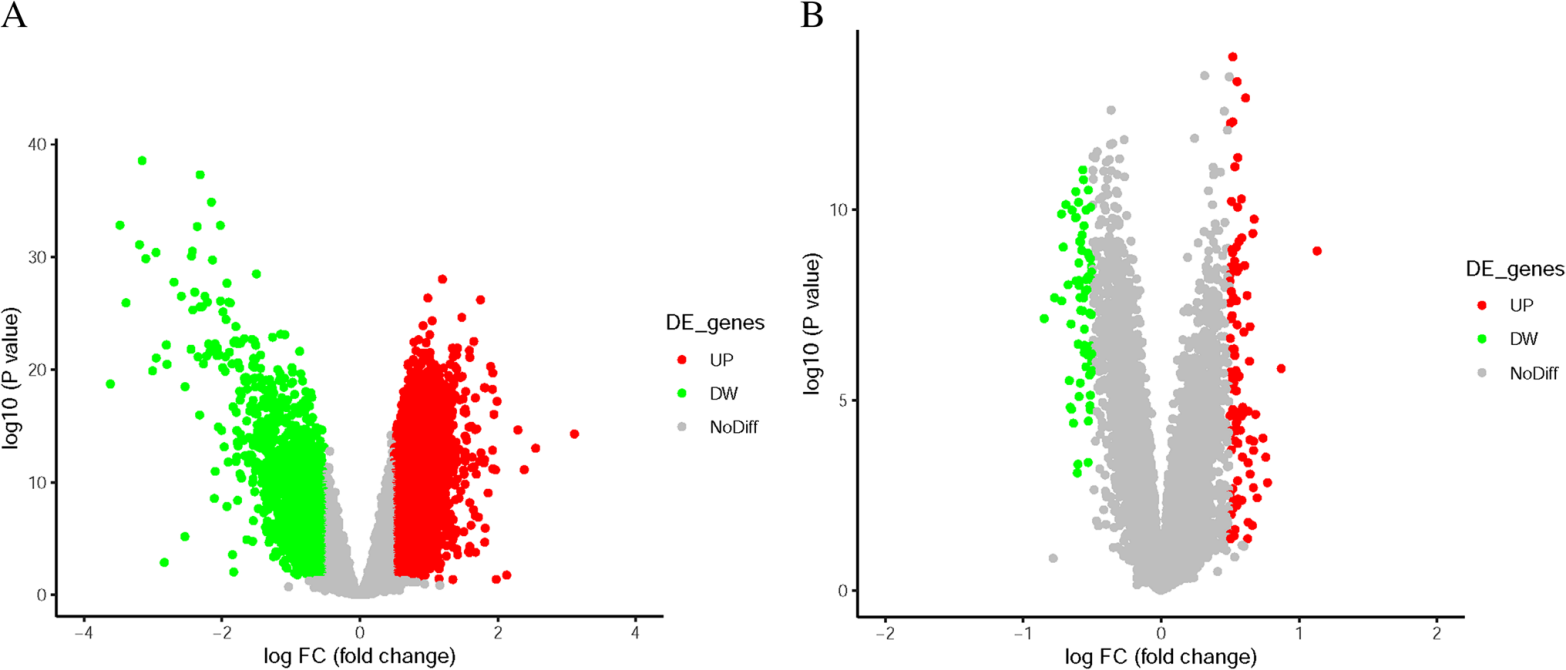
Volcano plot representing differential expression genes (DEGs) between control groups and IS groups (**A**, **B**) shows DEGs in GSE58294 and GSE16561 dataset, respectively

**Fig. 2:**
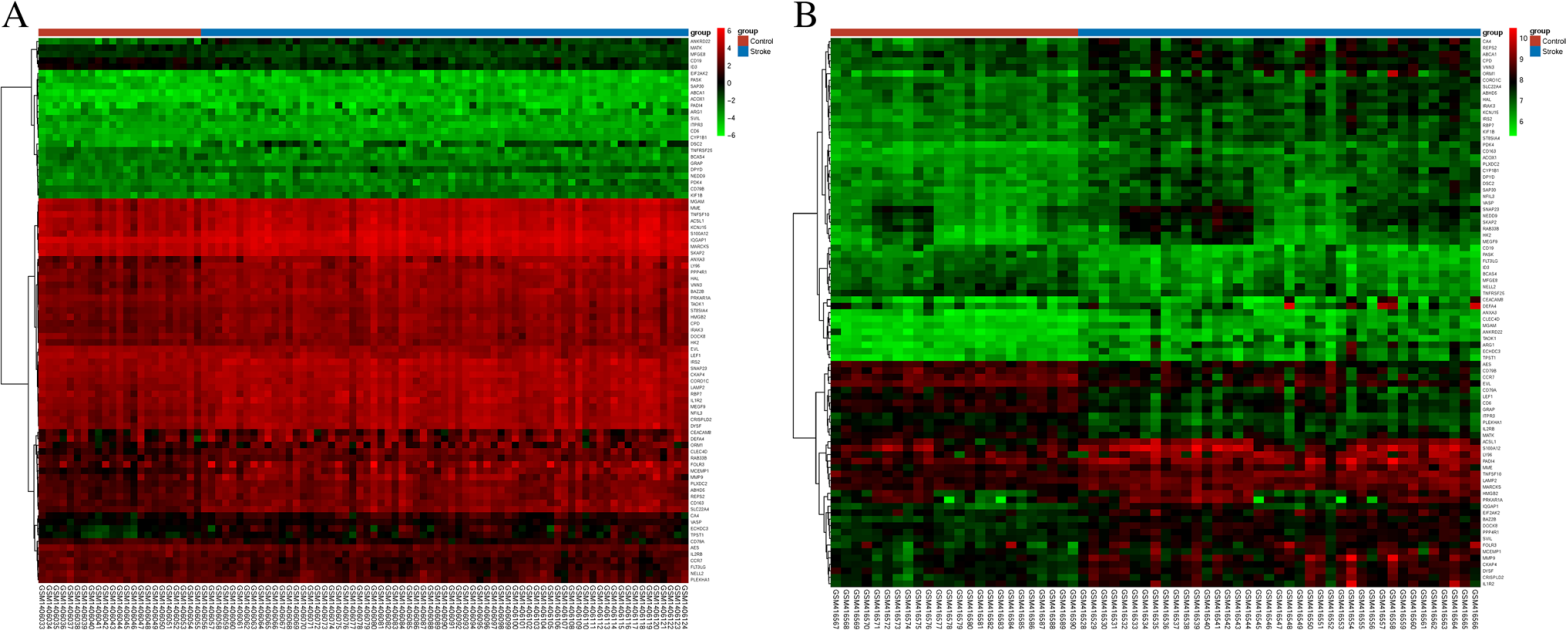
Blue indicatesthe stroke group; red indicates the control group. Red and green show differential gene expression in grouped samples; red indicates that the expression value is high; green indicates that the expression value is low (**A**, **B**). Shows heatmap in GSE58294 and GSE16561 respectively

According to the limiting conditions, the qualified gene chips were GSE58294 and GSE16561 in the GEO database including both controls and IS samples. Among them, GSE58294 chip belonged to GPL570 platform, including 23 control samples and 69 samples, while GSE16561 chip belonged to GPL6882, including 24 control samples and 39 IS samples. Sequencing information was obtained from human peripheral blood mononuclear cells. According to the criteria of *P* < 0.05 and |log_2_FC|≥ 0.5, 4898 DEGs in total were obtained from GSE58294, containing 3102 upregulated genes and 1796 downregulated genes. In the GSE16561 dataset, 169 DEGs were obtained, 97 of which were upregulated, and 72 of which were downregulated. The gene expression profile of each 2 of DEGs containing 2 sets of sample data was shown in Figs. 1 and 2.

**Fig. 3:**
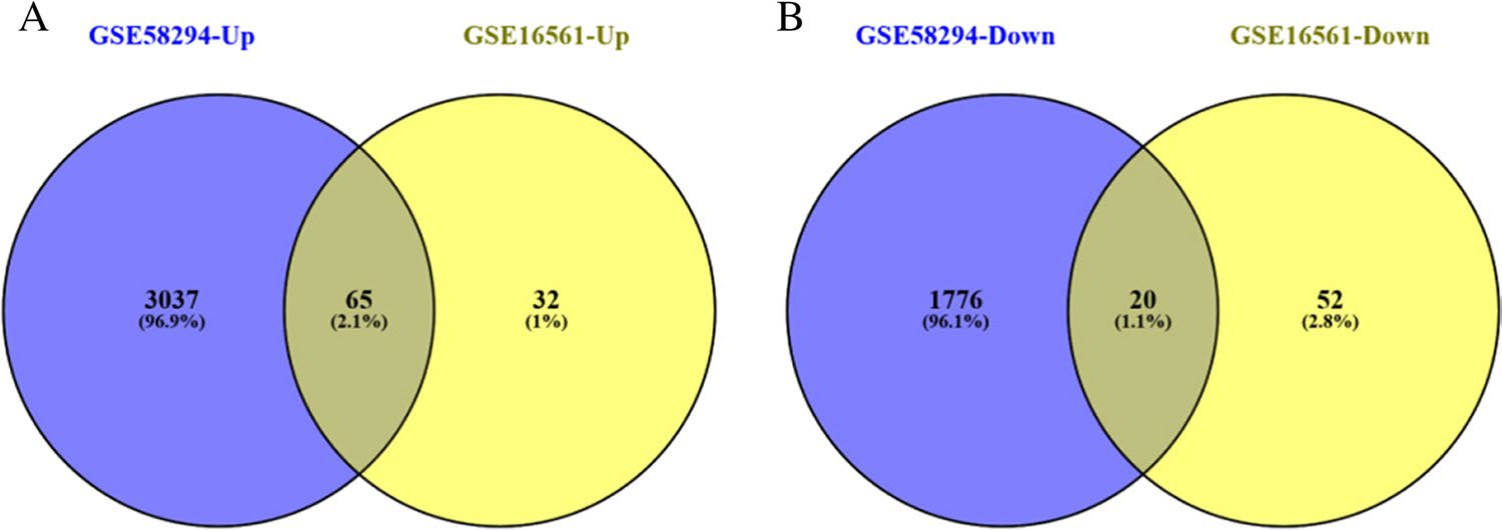
Venn diagrams showing the overlaps of numbers of DEGs between 2 selected GEO datasets (**A**, **B**) illustrate overlap of upregulated and downregulated genes in GSE58294 and GSE16561dataset, respectively

These genes were further filtered and then mapped by Venn diagram. As shown in Fig. 3, it was found that 85 genes were remarkably differentially expressed between the two groups, of which 65 genes were upregulated and 20 genes were downregulated.

### GO functional enrichment analysis and KEGG enrichment pathway

GO function enrichment analysis was carried out on 85 DEGs by David platform, and 56 GO items with significant differences were obtained, including 18 CC items, 27 BP items, and 11 MF items. The bubble chart showed the top 10 pathways.

**Fig. 4:**
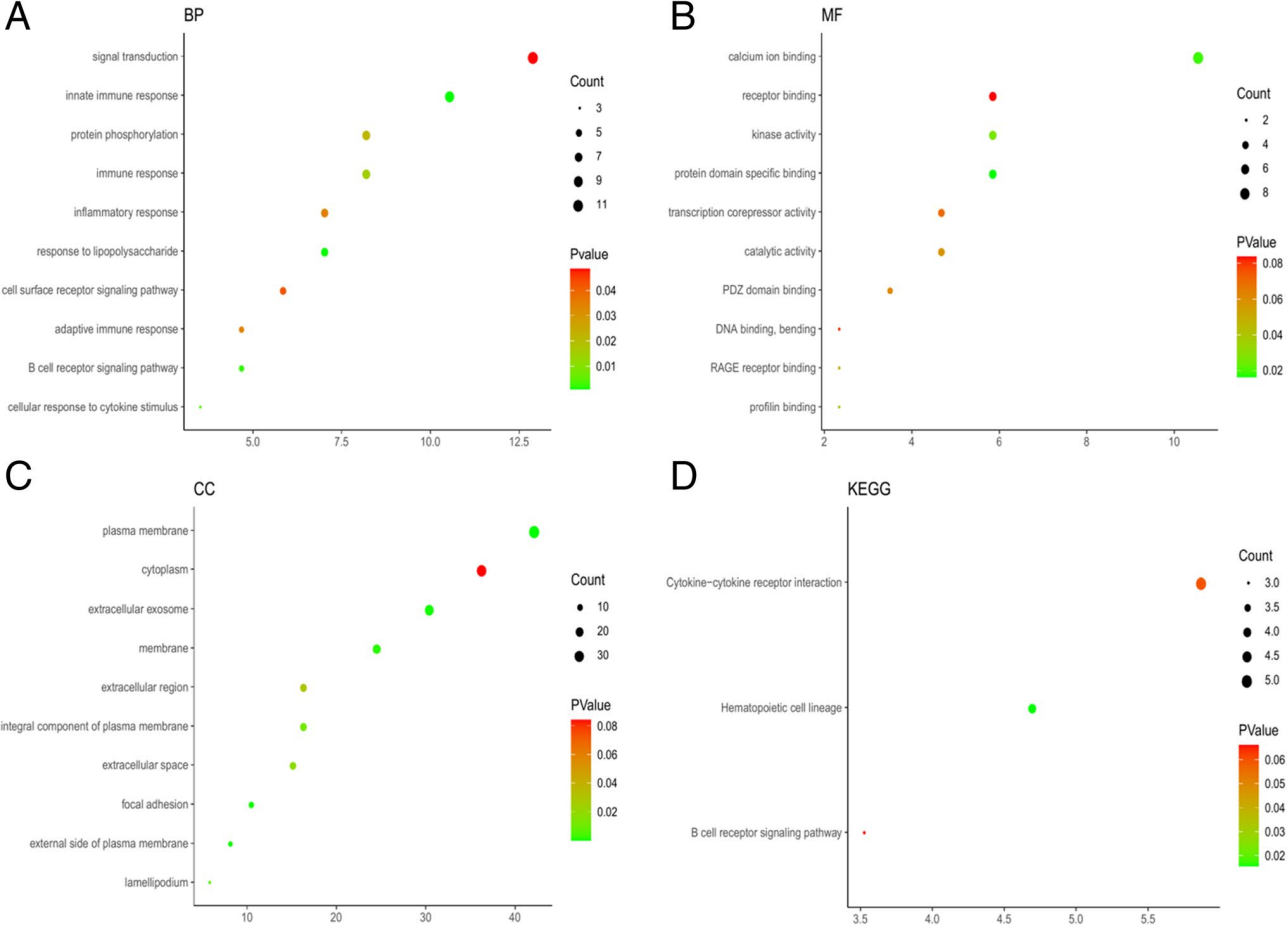
GO functional and KEGG pathway enrichment analysis of DEGs. GO functional analysis showing enrichment of DEGs in **A** biological process, **B** molecular function, **C** cellular component, **D** KEGG pathway enrichment analysis of DEGs

It can be seen from the graph that the biological processes of BP mediated by DEGs were mainly concentrated in signal transduction, innate immune response and protein phosphorylation. The results of CC were mainly concentrated in plasma membrane, cytoplasm, and extracellular exosomes. The results of MF showed that calcium ion binding, receptor binding, and kinase activity were important enrichment items. See Fig. 4 and Tables 2, 3, and 4 for detailed results.

**Table 2:** GO biological process terms for DEGs between the control and IS groups

GOTERM_BP	Term	PValue	Fold enrichment	Count	GeneRatio	Genes
1	GO:0,007,165 signal transduction	0.048507425	1.964164567	11	12.94117647	CD79B, PPP4R1, IL2RB, TNFSF10, NEDD9, FLT3LG, IRS2, TNFRSF25, IQGAP1, CORO1C, SKAP2
2	GO:0,045,087 innate immune response	0.001002881	4.339018088	9	10.58823529	CLEC4D, CD6, DEFA4, MATK, HMGB2, EIF2AK2, S100A12, LY96, PADI4
3	GO:0,006,955 immune response	0.015163648	3.44693704	7	8.235294118	CD79B, NFIL3, IL1R2, TNFSF10, CCR7, TNFRSF25, CEACAM8
4	GO:0,006,468 protein phosphorylation	0.02156091	3.182369504	7	8.235294118	PPP4R1, TAOK1, MATK, PDK4, EIF2AK2, IRAK3, PASK
5	GO:0,032,496 response to lipopolysaccharide	0.001113916	7.584462511	6	7.058823529	CD6, HMGB2, LY96, CCR7, IRAK3, TNFRSF25
6	GO:0,006,954 inflammatory response	0.034625375	3.281931008	6	7.058823529	TPST1, ORM1, S100A12, LY96, CCR7, TNFRSF25
7	GO:0,007,166 cell surface receptor signaling pathway	0.041831035	3.783004416	5	5.882352941	CD19, TNFSF10, LY96, EVL, TNFRSF25
8	GO:0,050,853 B cell receptor signaling pathway	0.002168409	15.3561957	4	4.705882353	CD79B, CD79A, PLEKHA1, CD19
9	GO:0,002,250 adaptive immune response	0.033682623	5.60293627	4	4.705882353	CD79B, CD79A, CLEC4D, CD6
10	GO:0,071,345 cellular response to cytokine stimulus	0.004867389	28.26936027	3	3.529411765	MME, LEF1, CCR7

**Table 3:** GO molecular function terms for DEGs between the control and IS groups

GOTERM_MF	Term	PValue	Fold enrichment	Count	GeneRatio	Genes
1	GO:0,005,509 calcium ion binding	0.018741709	2.648692469	9	10.58823529	NELL2, ANXA3, REPS2, DYSF, S100A12, ITPR3, PADI4, IQGAP1, DSC2
2	GO:0,019,904 protein domain-specific binding	0.016406406	5.072415865	5	5.882352941	LAMP2, HMGB2, ID3, IRS2, IQGAP1
3	GO:0,016,301 kinase activity	0.026474471	4.377852697	5	5.882352941	NELL2, PRKAR1A, TAOK1, PDK4, FLT3LG
4	GO:0,005,102 receptor binding	0.083617609	2.988845609	5	5.882352941	ABCA1, ACOX1, MATK, TNFSF10, FLT3LG
5	GO:0,003,824 catalytic activity	0.058139482	4.48962766	4	4.705882353	HAL, MGAM, ACSL1, ECHDC3
6	GO:0,003,714 transcription corepressor activity	0.069792981	4.157881773	4	4.705882353	NFIL3, ID3, SAP30, AES
7	GO:0,030,165 PDZ domain binding	0.061420984	7.360901163	3	3.529411765	SLC22A4, PLEKHA1, ACOX1
8	GO:0,005,522 profilin binding	0.041348098	46.89166667	2	2.352941176	VASP, EVL
9	GO:0,050,786 RAGE receptor binding	0.050304763	38.36590909	2	2.352941176	HMGB2, S100A12
10	GO:0,008,301 DNA binding, bending	0.081007285	23.44583333	2	2.352941176	LEF1, HMGB2

**Table 4:** GO cellular component terms for DEGs between the control and IS group

GOTERM_CC	Term	*P* value	Fold enrichment	Count	GeneRatio	Genes
1	GO:0,005,886 plasma membrane	5.44E-05	1.895240406	36	42.35294118	SLC22A4, SNAP23, DYSF, LY96, IRS2, ITPR3, IQGAP1, CD79B, CD79A, CD19, LAMP2, CA4, S100A12, CCR7, ABCA1, VASP, SVIL, MGAM, CD163, PLEKHA1, MME, ACSL1, ANXA3, IL1R2, KCNJ15, CKAP4, MARCKS, CLEC4D, ACOX1, VNN3, CPD, IL2RB, TNFRSF25, CEACAM8, DSC2, SKAP2
2	GO:0,005,737 cytoplasm	0.08417535	1.287921067	31	36.47058824	SNAP23, LEF1, HMGB2, NEDD9, ITPR3, ABHD5, IQGAP1, CORO1C, CD79B, CD79A, REPS2, S100A12, VASP, SVIL, PLEKHA1, MME, ANXA3, ARG1, IL1R2, EIF2AK2, IRAK3, PASK, RBP7, PRKAR1A, TAOK1, DPYD, ID3, EVL, PADI4, GRAP, SKAP2
3	GO:0,070,062 extracellular exosome	5.30E-04	2.006674459	26	30.58823529	ORM1, SNAP23, DYSF, IQGAP1, CD79B, CD19, LAMP2, CA4, TNFSF10, VASP, MGAM, PLEKHA1, MME, ANXA3, ARG1, PLXDC2, MMP9, CKAP4, RAB33B, MARCKS, CRISPLD2, TAOK1, CPD, CEACAM8, MFGE8, DSC2
4	GO:0,016,020 membrane	0.001726377	2.070909091	21	24.70588235	CD163, ACSL1, ANXA3, DOCK8, KCNJ15, EIF2AK2, FLT3LG, ITPR3, CKAP4, HK2, TPST1, CD6, PRKAR1A, ACOX1, CPD, IL2RB, LAMP2, CA4, EVL, FOLR3, MFGE8
5	GO:0,005,887 integral component of plasma membrane	0.011315379	2.146525324	14	16.47058824	ABCA1, SLC22A4, CD163, MME, KCNJ15, ITPR3, CD79B, CD79A, CD6, CD19, IL2RB, TNFSF10, TNFRSF25, CEACAM8
6	GO:0,005,576 extracellular region	0.029681411	1.886542443	14	16.47058824	ORM1, CD163, DEFA4, IL1R2, FLT3LG, MMP9, NELL2, CD6, CRISPLD2, TNFSF10, S100A12, TNFRSF25, FOLR3, MFGE8
7	GO:0,005,615 extracellular space	0.018583106	2.093824018	13	15.29411765	ORM1, ARG1, DEFA4, HMGB2, FLT3LG, LY96, MMP9, VNN3, CPD, LAMP2, TNFSF10, CEACAM8, MFGE8
8	GO:0,005,925 focal adhesion	4.05E-04	4.99378882	9	10.58823529	VASP, SVIL, MARCKS, MME, SNAP23, NEDD9, EVL, IQGAP1, CORO1C
9	GO:0,009,897 external side of plasma membrane	4.24E-04	7.129890454	7	8.235294118	ABCA1, CD79B, CD79A, CD19, IL2RB, CCR7, MFGE8
10	GO:0,030,027 lamellipodium	0.006143732	6.779761905	5	5.882352941	VASP, DYSF, NEDD9, EVL, CORO1C

The results of KEGG enrichment pathway showed that DEGs was mainly involved in cytokine–cytokine receptor interaction, hematopoietic cell lineage and B cell receptor signaling pathway. See Fig. 4 and Table 5 for the results.

**Table 5:** KEGG pathway enrichment terms for DEGs between the control and IS group

KEGG_pathway	Term	PValue	Fold enrichment	Count	GeneRatio	Genes
1	hsa04060 Cytokine-cytokine receptor interaction	0.059651063	3.291702555	5	5.882352941	IL1R2, IL2RB, TNFSF10, CCR7, TNFRSF25
2	hsa04640 Hematopoietic cell lineage	0.01572673	7.355252606	4	4.705882353	MME, CD19, IL1R2, FLT3LG
3	hsa04662 B cell receptor signaling pathway	0.066036489	6.955510617	3	3.529411765	CD79B, CD79A, CD19

### Construction of PPI network and identification of hub genes

**Fig. 5:**
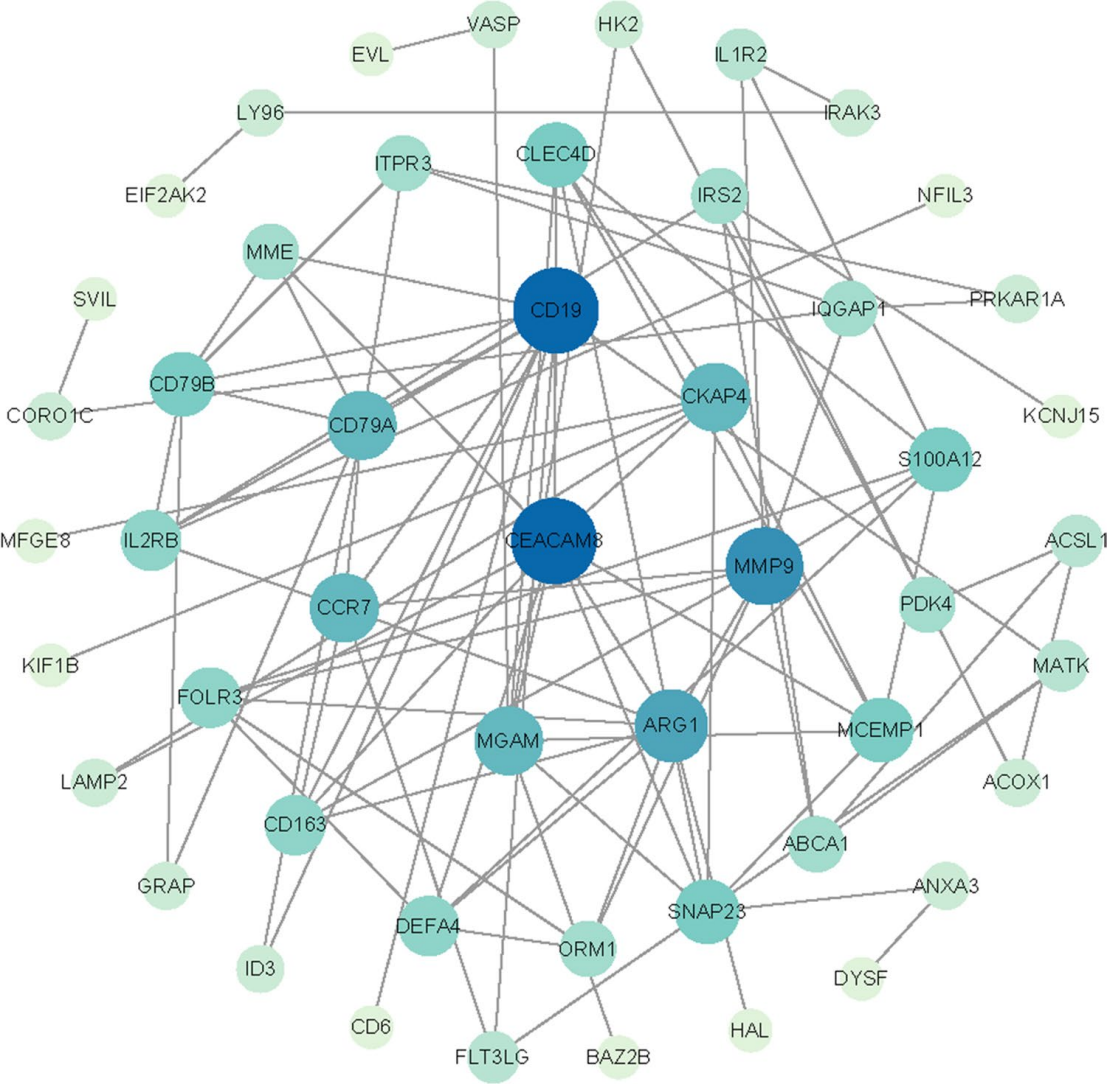
Protein–protein interaction network of 65 upregulated and 20 downregulated genes were analyzed using Cytoscape software. The edges between 2 nodes represent the gene–gene interactions. The size and color of the nodes corresponding to each gene were determined according to the degree of interaction. The closer to the blue node, the higher connectivity between 2 nodes

**Fig. 6:**
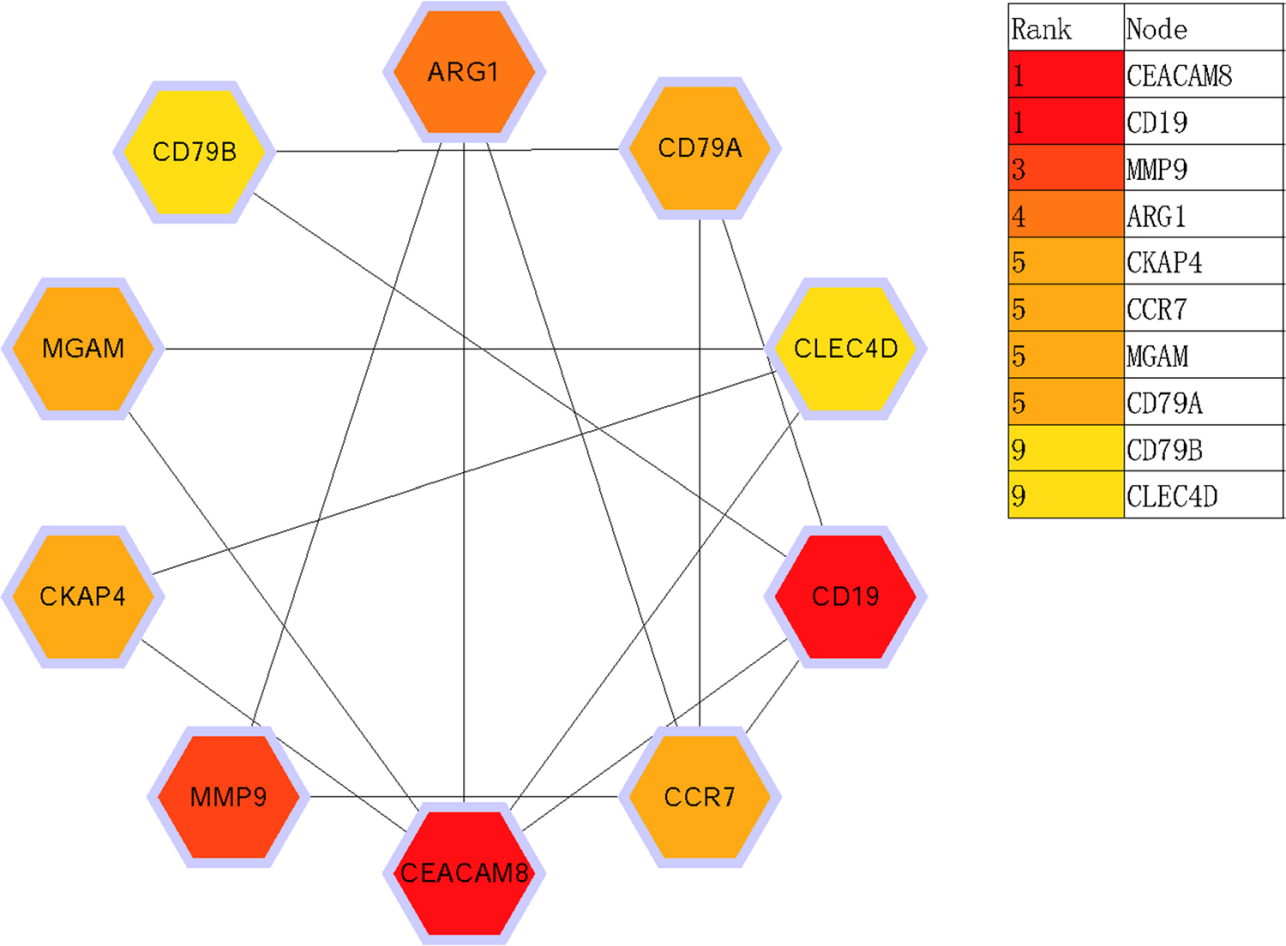
Protein–protein interaction network for the top 10 hub genes. Node color indicates the number of degrees. The top 10 ranked hub genes are depicted using a pseudocolor scale. Red color stands for highest degree, and yellow color represents lowest degree

PPI network of DEGs was constructed by STRING v10, and visualized by Cytoscape, the result was shown in the Fig. 5. CytoHubba of Cytoscape was used to determine the key nodes in PPI network, ten key genes were obtained, namely CEACAM8, CD19, MMP9, ARG1, CKAP4, CCR7, MGAM, CD79B, and CLEC4D. According to the node degree score generated by Cytoscape, the potential hub genes were determined see Fig. 5. The results showed that CEA cell adhesion molecule 8 (CEACAM8, score 11) and CD19 molecule (CD19, score 11) were the most significant genes. The rest were matrix metalloproteinases 9 (MMP9, score 9), arginase 1 (ARG1, score 8), C–C chemokine receptor 7 (CCR7, score 8), maltase-glucoamylase (score 7), CD79a molecule (CD79A, score 7), CD79B molecule (CD79B, score 6), and CLEC4D (score 6), see Fig. 6.

### Integrated miRNA/gene regulatory networks

**Fig. 7:**
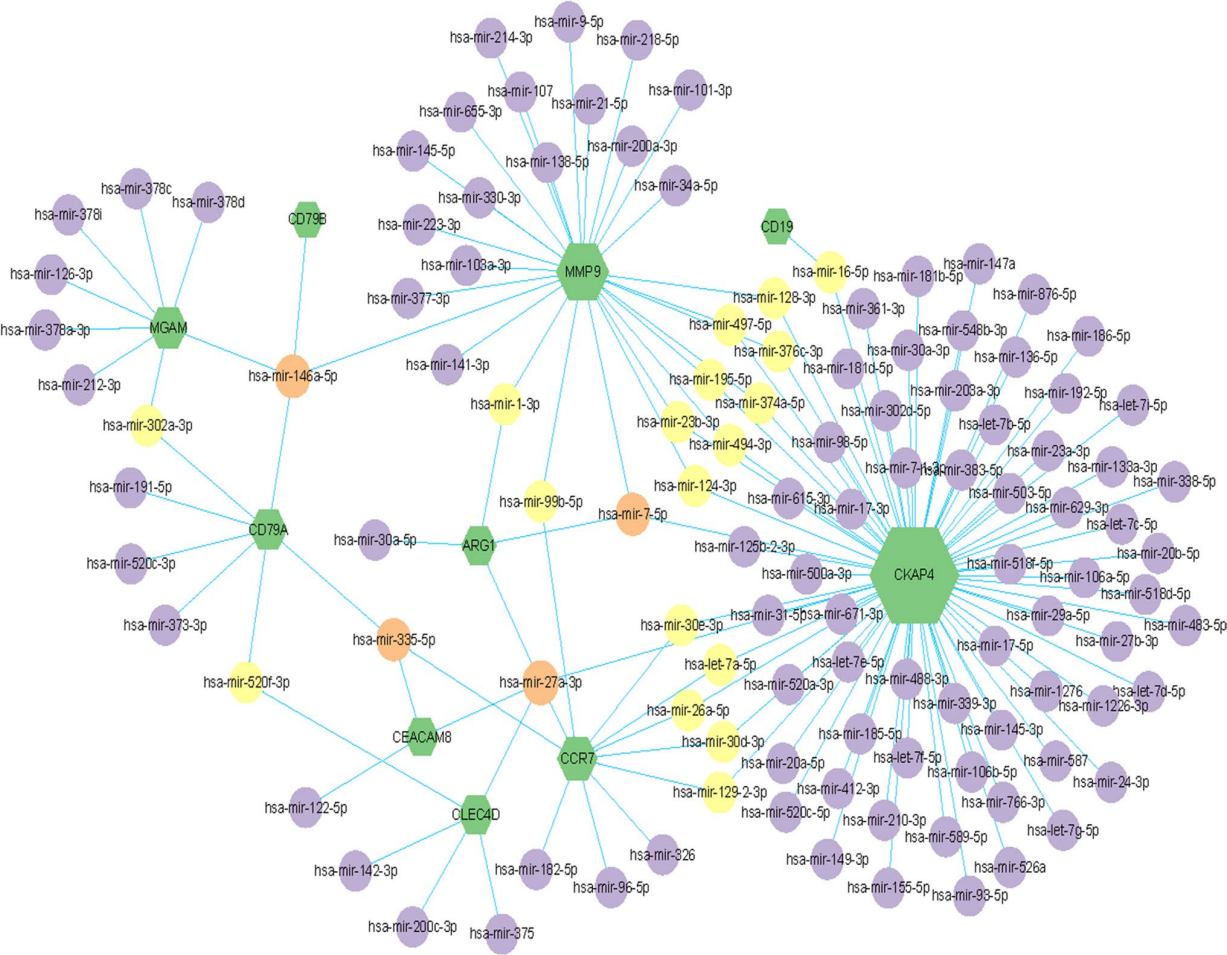
Integrated miRNA-DEGs networks for the top 10 hub genes. Green hexagons represent 10 hub genes. Red circles represent miRNA which has high connectivity with hub genes, yellow circles represent miRNA which has moderate connectivity with hub genes, purple circles represent miRNA which has low connectivity with hub genes

The significant difference miRNA-gene regulatory network was constructed by Cytoscape software, and the target miRNAs were predicted according to the network analysis database. The top 10 DEGs and their corresponding regulatory miRNAs molecules were shown in the Fig. 7. In 10 DEGs, for example, MMP9, CD79B, MGAM, and CD79A could be used as common targets for predicting hsa-mir-146a-5p. MMP9, CKAP4, and ARG1 could be used as common targets for predicting hsa-mir-7-5p. Common targets of hsa-mir-335-5p were CD79A, CEACAM8, and CCR7. Common targets of hsa-mir-27a-3p were ARG1, CKAP4, CEACAM8, CCR7, and CLEC4D. However, these findings need to be further verified in future studies.

## Discussion

The incidence of IS is caused by a combination of many factors, such as environment and heredity. In this study, the gene chip information of peripheral blood mononuclear cells of IS patients and healthy controls was obtained by using GEO database, and 85 DEGs were analyzed and screened by using GEO2R software, including 65 upregulated genes and 20 downregulated genes. Then, GO function enrichment analysis and KEGG pathway enrichment analysis were carried out on the DEGs obtained. The results of GO functional classification showed that DEGs mainly concentrates on signal transduction, immune response, inflammatory response, and receptor binding. In the PPI network of DEGs, the scores of CEACAM8, CD19, and MMP9 were higher. The results of KEGG pathway enrichment analysis showed that DEGs mainly mediated cytokine-receptor interaction, hematopoietic pathway, and B cell receptor signaling pathway.

Adhesion molecule is a kind of membrane surface glycoprotein which can mediate the adhesion between cell–cell and cell-extracellular matrix. It is mainly expressed in leukocytes, platelets, and endothelial cells. There are many kinds of adhesion molecules including platelet membrane glycoprotein [11]. At present, the view that inflammation and immune response play a key role in cerebrovascular diseases has been widely recognized. Inflammatory cells can lead to the formation of early pathological changes of cerebrovascular diseases, inflammatory effector molecules can lead to the progression of pathological changes, and inflammatory activation can lead to the occurrence of acute ischemic cerebrovascular diseases. The adhesion of circulating leukocytes to endothelial cells and migration into arterial walls is an early step in the formation of atherosclerosis, which needs to be mediated by cell adhesion molecules expressed on the surface of vascular endothelial cells. Adhesion molecules allow monocytes and lymphocytes to roll, adhere tightly, and migrate across endothelium. Adhesion molecules play an important role in the occurrence of stroke and cerebral ischemia–reperfusion (I/R) injury. The adhesion in vascular endothelial cells and transmembrane migration of leukocytes need to be mediated by adhesion molecules, so adhesion molecules are an important inflammatory mediator. In addition, platelet activation is also related to adhesion molecules. At present, it is considered that cerebral I/R injury is essentially an inflammatory process, in which white blood cells infiltrate into brain tissue through the interaction with adhesion molecules. Therefore, the treatment measures related to anti-adhesion molecules were expected to become a new field in the treatment of cerebrovascular diseases [12, 13]. In addition, adhesion molecules can reflect whether vascular endothelial cells are damaged or not, and can also reflect the activation state of leukocytes and platelets, which all play a role in the occurrence and development of cerebrovascular diseases. Therefore, adhesion molecules can be used as predictors of stroke in theory. The correlation between adhesion molecules and cerebrovascular diseases has been preliminarily confirmed, and the determination of adhesion molecules may have potential value in preventing and monitoring cardiovascular and cerebrovascular diseases, guiding clinical treatment.

CD19 is a member of B-cell-specific immunoglobulin superfamily, which is expressed by early B cells during the period from heavy chain rearrangement to plasma cell differentiation. CD19 has been reported to enhance the activity of Src family protein tyrosine kinase and mitogen-activated protein kinase, promote cell proliferation, and positively regulate the function of B cells. Its functional diversity and important signal transduction make it play an important role. If abnormal expression occurs, it will lead to B cell–related diseases [14]. In addition, it can mediate the pathophysiological process of diseases by affecting immune function.

Matrix metalloproteinase families (MMPs) are closely related to cerebrovascular diseases. Some studies have shown that [15] there is a certain relationship between acute ischemic stroke and MMPs. By detecting patients with acute ischemic stroke, it is found that the level of MMPs in serum will obviously increase. Elevated MMPs can degrade extracellular matrix, aggravate vascular brain edema, and damage neurons. The experiment found that [16] knocking out MMPs gene or adding MMPs inhibitor can obviously reduce the injury of stroke. MMP9 is one of the most closely related members of MMPs family with cerebrovascular diseases, which can degrade the components of cerebrovascular basement membrane, increase the permeability of blood–brain barrier, and aggravate the occurrence of cerebral edema after being activated. The research also confirmed that the high expression of MMP9 can aggravate the destruction of blood–brain barrier and promote the occurrence of brain edema, and the purpose of relieving brain edema can be achieved by knocking out MMP9 in mice or using MMPs inhibitors [17]. In the central nervous system diseases such as inflammation, MMP9 could lead to cerebral hemorrhage and brain edema by breaking the blood–brain barrier, and had toxic and side effects on neurons in varying degrees [18]. The results of clinical research on patients with cerebral ischemia suggested that the expression level of MMP9 in human cerebral ischemia patients was higher than that in healthy people [19], and the expression level of MMP9 was also closely related to the prognosis of patients with cerebral ischemia. There is a certain relationship between the expression level of MMP9 and the hemorrhagic transformation after cerebral ischemia, the expression level of MMP9 can predict the possibility of transforming into cerebral hemorrhage. After later observation, it was found that the plasma level of MMP9 was higher in patients with hemorrhagic transformation after cerebral hemorrhage. After cerebral ischemia, the degranulation of neutrophils leads to the release of stored MMP9, which leads to the increase of blood MMP9 level. Therefore, MMP9 can be used as a blood detection marker in the prediction of hemorrhagic transformation of patients, and the high expression level of MMP9 after cerebral ischemia is more likely to lead to hemorrhagic transformation after ischemia [20]. The disorder of MMP9 gene level may participate in the pathophysiological process of stroke, and may become a new molecular target for diagnosis and prognosis of stroke patients.

The results of GO functional enrichment analysis showed that the biological processes involved in DEGs were mainly concentrated in signal transduction, immune response, inflammatory response, and cytokine-receptor interaction, suggesting that inflammatory response played an important role in the occurrence and development of IS. Pathological inflammation can cause permanent damage to functional cells and vegetative cells in the brain, and the damage caused by IS could be alleviated by inhibiting inflammation [21]. In the rat model of middle cerebral artery embolism, electroacupuncture treatment can inhibit the pathological inflammatory reaction mediated by NLRP3 inflammatory corpuscles through the pathway mediated by α7 nicotinic acetylcholine receptor, thus alleviating the brain injury induced by IS [22]. This prediction result of GO functional enrichment analysis was consistent with the results of many clinical studies, which indicated that bioinformatics analysis could provide new ideas for the prevention and clinical treatment of IS [23]. Many animal experiments and clinical studies have shown that inflammatory response and immune response played an important role in the early stage after stroke, and affected the prognosis and treatment of stroke [24–27].

Ten key genes in the pathogenesis of IS were identified by using the STRING software of Cytoscape. Most of these key genes were related to inflammatory response, which was the core link of IS injury. Studies had shown [28] that abnormal expression of inflammation-related genes was the genetic basis of IS, and targeted therapy for these genes may provide a new direction for the prevention and treatment of IS. The occurrence and development of IS is mediated by multiple genes [29]. KEGG pathway analysis showed that multiple DEGs mediated the same information transmission pathway, PPI network results also showed that multiple DEGs were closely related, and miRNA results also showed that multiple key genes could predict common target miRNA. Recent studies had found that miRNAs were involved in the pathological and physiological processes of many diseases, including tumors, immune system, cell proliferation, cardiovascular diseases, and nervous system diseases [30]. Stroke affected the level of miRNA in brain and circulation. MiRNA played a key role in the pathogenesis of stroke and its complications, and participated in the regulation of key metabolism, inflammation, and angiogenesis [31]. Studies have shown that miRNAs played a key role in regulating cell growth, differentiation, progression, and apoptosis, as well as neuron development, hematopoiesis, and repair and remodeling of injured tissues [32]. MiRNA-146a is a strong pro-apoptosis factor [33]. As an important inflammatory microRNA, miR-146a can be found in both immune cells and circulating cells [34]. At the level of translation inhibition, miR-146a can act as a negative regulator of inflammation, mediating IL-1 signaling pathway of IRAK1 [35]. In addition, the animal I/R model study explored the relationship between miR-146a and myocardial I/R injury in mice heart by upregulating the expression of miR-146a, and confirmed that miR-146a can protect myocardium from I/R injury [36]. MiRNAs can mediate the pathological and physiological processes of stroke and its complications. Studies have shown that miRNAs is promising as a treatment for stroke and its complications [37]. Samaraweera et al. [38] found that miR-335 can downregulate the expression level of HAND1 and JAG1, block the growth and differentiation of neurons, and participate in the regulation of neuronal development. Tom et al. [39] found that overexpression of miR-335 can downregulate the expression of AP-1 which is related to cell proliferation, differentiation, and apoptosis. These studies suggest that miR-335 may be an important neuronal regulatory factor, and the abnormal expression of miR-335 may be closely related to nervous system diseases. Our current results indicated that some miRNAs including mir-146a, mir-335, mir-27a, and mir-7 may play a key role in the occurrence and development of IS diseases, and the role of these miRNAs in IS may need further discussion. In addition, the research on stroke genes and miRNAs is still limited.

Gene regulatory network plays an important role in the pathophysiological process of stroke. This discovery will help us better understand the pathogenesis of stroke and provide effective and novel treatment strategies for stroke. However, our research also has some limitations, such as the following: (1) Our current research only involves the top 10 hub genes; (2) the specific molecular mechanism of hub genes and miRNAs in stroke regulation is insufficient; (3) in the constructed network, there is a lack of research on the functions of hub genes and miRNA.

## Conclusion

Our findings suggested that compared with the healthy control group, the expressions of CEACAM8, CD19, MMP9, ARG1, CKAP4, CCR7, MGAM, CD79B, and CLEC4D in patients with IS were significantly upregulated, which may have an important influence on the pathophysiological mechanism of ischemic stroke. Some potential target miRNAs such as hsa-mir-146a-5p, hsa-mir-7-5p, hsa-mir-335-5p, and hsa-mir-27a-3p were also predicted. Identification of these genes and miRNAs may contribute to the development of early diagnostic strategies, prognostic markers, and therapeutic targets for IS. However, experimental research is still necessary to validate the functions of these molecules in IS.

## Data Availability

The data used in this study were downloaded from the GEO database. The data used to support the findings of this study are available from corresponding websites upon request.

## References

[1] Favate AS, Younger DS (2016). Epidemiology of ischemic stroke. Neurol Clin.

[2] Seidkhani-Nahal A, Khosravi A, Mirzaei A (2021). Serum vascular endothelial growth factor (VEGF) levels in ischemic stroke patients: a systematic review and meta-analysis of case–control studies. Neurol Sci.

[3] Yang X, Hongyang Xu, Xiaoshan Du et al (2019) Association between miR- 146a gene rs2910164 polymorphism and risk of ischemic stroke in Asians population: a meta-analysis. Shanxi Med J 48(8):883–887

[4] Zhang S, Cheng S, Zhang Z (2021). Related risk factors associated with post-stroke fatigue: a systematic review and meta-analysis. Neurol Sci.

[5] Pei L, Cai Y, Zhang Y, Ke X (2019). Transcriptome sequencing unravels potential biomarkers at different stages of cerebral ischemic stroke. Front genet.

[6] Li Hong, Shasha Yu, Wang Rui (2017). Genetic variant of kalirin gene is associated with ischemic stroke in a Chinese Han population. BioMed Res Int.

[7] Li S, Ning Lu, Li Z, Jiao B (2017). Adiponectin gene poly-morphism and ischemic stroke subtypes in a Chinese population. J Stroke Cerebrovasc Dis.

[8] Hernández M, Quijada NM, Rodríguez-Lázaro D, Eiros JM (2020). Bioinformatics of next generation sequencing in clinical microbiology diagnosis. Rev Argent Microbiol.

[9] Ogata H, Goto S, Sato K, Fujibuchi W, Bono H, Kanehisa M (1999). KEGG: Kyoto encyclopedia of genes and genomes. Nucleic Acids Res.

[10] Ashburner M, Ball CA, Blake JA, Botstein D, Butler H, Cherry JM (2000). Gene ontology: tool for the unification of biology The gene ontology consortium. Nat Genet.

[11] Terao S, Yilmaz G, Stokes KY, Ishikawa M, Takeshi Kawase D, Granger N (2008). Inflammatory and injury responses to ischemic stroke in obese mice. Comparative Study.

[12] Rubio-Guerra AF, Vargas-Robles H, Serrano AM, Lozano-Nuevo JJ, Escalante-Acosta BA (2009). Correlation between the levels of circulating adhesion molecules and atherosclerosis in type-2 diabetic normotensive patients: circulating adhesion molecules and atherosclerosis. Cell Adh Migr.

[13] Sierakowska-Fijałek A, Baj Z, Kaczmarek P, Stepień M, Rysz J (2008). Estimation of relation between homocysteine concentration and selected lipid parameters and adhesion molecules concentration in children with atherosclerosis risk factors. Pol Merkur Lekarski.

[14] Li X, Ding Y, Zi M, Sun Li, Zhang W, Chen S, Yuekang Xu (2017). CD19, from bench to bedside. Immunol Lett.

[15] Kucera R, Smid D, Topolcan O (2016). Searching for new biomarkers and the use of multivariate analysis in gastric cancer diagnostics. Anticancer Res.

[16] Bae M-J, Karadeniz F, Lee S-G, Seo Y, Kong C-S (2016). Inhibition of MMP-2 and MMP-9 activities by limonium tetragonum extract. Prev Nutr Food Sci.

[17] He Wu, Zhang Z, Li Y, Zhao R, Li H, Song Y, Qi J, Wang J (2010). Time course of upregulation of inflammatory mediators in the hemorrhagic brain in rats: correlation with brain edema. Neurochem Int.

[18] Yang R, Zhang Y, Huang D (2017). Miconazole protects blood vessels from MMP9 - dependent rupture and hemorrhage. Dis Model Mech.

[19] Wang TH, Xiong LL, Yang SF (2016). LPS pretreatment provides neuroprote-ctive roles in rats with subarachnoid hemorrhage by downregulating MMP9 and Caspase3 associated with TLR4 signaling activation. Mol Neurobiol.

[20] Yang X, Wang G (2019) MMP9 advances in brain isoemia. Guangdong Med J 40(6):875–878

[21] Ai QD, Chen C, Chu S (2019). IMM-H004 therapy for permanent focal ischemic cerebral injury via CKLF1/CCR4-mediated NLRP3 inflammasome activation. Transl Res.

[22] Jiang T, Wu M, Zhang Z (2019). Electroacupuncture attenuated cerebral ischemia injury and neuroinflammation through α7nAChR - mediated inhibition of NLRP3 inflammasome in stroke rats. Mol Med.

[23] Chen A, Xu Y, Yuan J (2018). Ginkgolide B ameliorates NLRP3 inflammasome activation after hypoxic - ischemic brain injury in the neonatal male rat. Int J Dev Neurosci.

[24] Amadatsu T, Morinaga J, Kawano T, Oike Y (2016). Macrophage-derived angiopoietin-like protein 2 exacerbates brain damage by accelerating acute inflammation after ischemia-reperfusion. PLoS One.

[25] Lin R, Cai J, Kostuk EW (2016). Fumarate modulates the immune /inflammatory response and rescues nerve cells and neurological function after stroke in rats. J Neuroinflammation.

[26] Molnar T, Pusch G, Nagy L (2016). Correlation of the larginine pathway of the L-arginine pathway with thrombo-inflammation may contribute to the out-come of acute ischemic stroke. J Stroke Cerebrovasc Dis.

[27] Rodriguez-Grande B, Swana M, Nguyen L (2014). The acutephase protein PTX3 is an essential mediator of glial scar formation and resolution of brain edema after ischemic injury. J Cereb Blood Flow Metab.

[28] Guo H, Callaway JB, Ting JP (2015). Inflammasomes: mechanism of action, role in disease, and therapeutics. Nat Med.

[29] Chatterjee S, Ahituv N (2017). Gene regulatory elements, major drivers of human disease. Annu Rev Genomics Hum Genet.

[30] Volny O, Kasickova L, Coufalova D (2015). MicroRNAs in cerebrovascular disease. Adv Exp Med Biol.

[31] Yuan T, Yang T, Chen H, Fu D, Hu Y, Wang J, Yuan Q, Yu H, Xu W, Xie X (2019). New insights into oxidative stress and inflammation during diabetes mellitus-accelerated atherosclerosis. Redox Biol.

[32] Garzon R, Fabbri M, Cimmino A, Calin GA, Croce CM (2006). MicroRNA expression and function in cancer. TRENDS Mol Med.

[33] Sha M, Ye J, Zhang L-X, Luan Z-Y, Chen Y-B (2013). Celastrol induces apoptosis of gastric cancer cells by miR-146a inhibition of NFkappaB activity. Cancer Cell Int.

[34] Prattichizzo F, Bonafe M, Ceka A (2016). Endothelial cell senescence and inflammaging: microRNAs as biomarkers and innovative therapeutic tools. Curr Drug Targets.

[35] Bhaumik D, Scott GK, Schokrpur S, Patil CK (2009). MicroRNAs miR-146a/b negatively modulate the senescence-associated inflammatory mediators IL-6 and IL-8. Aging (AlbanyNY).

[36] Wang X, Ha T, Liu Li, Zou J, Zhang X (2013). Increased expression of microRNA-146a decreases myocardial ischaemia/reperfusion injury. Cardiovasc Res.

[37] Chen J, Cui C, Yang X, Xu J (2017). MiR-126 affects brain-heart interaction after cerebral ischemic stroke. Transl Stroke Res.

[38] Samaraweera L, Grandinetti KB, Huang R, Spengler BA, Ross RA (2014). MicroRNAs define distinct human neuroblastoma cell phenotypes and regulate their differentiation and tumorigenicity and regulate their differentiation and tumorigenicity. BMC Cancer.

[39] Tomé M, Sepúlveda JC, Delgado M (2014). miR-335 correlates with senescence/aging in human mesenchymal stem cells and inhibits their therapeutic actions through inhibition of AP-1 activity. Stem Cells.

